# Insights into the Diversity and Population Structure of Predominant *Typhlocybinae* Species Existing in Vineyards in Greece

**DOI:** 10.3390/insects14110894

**Published:** 2023-11-19

**Authors:** Vasiliki Evangelou, Ioanna Lytra, Afroditi Krokida, Spyridon Antonatos, Iro Georgopoulou, Panagiotis Milonas, Dimitrios P. Papachristos

**Affiliations:** Scientific Directorate of Entomology and Agricultural Zoology, Benaki Phytopathological Institute, 8 Stefanou Delta Str., Kifissia, 14561 Athens, Greece; i.lytra@bpi.gr (I.L.); a.krokida@bpi.gr (A.K.); s.antonatos@bpi.gr (S.A.); i.georgopoulou@bpi.gr (I.G.); p.milonas@bpi.gr (P.M.); d.papachristos@bpi.gr (D.P.P.)

**Keywords:** *Typhlocybinae*, vineyards, distribution, fauna, *Hebata* sp., barcoding, systematics, Greece

## Abstract

**Simple Summary:**

Leafhoppers are tiny insects that are found in large populations in many different crops. They feed on the leaves and can transmit pathogens at the same time, damaging the plants and reducing final production. Several species are found in vineyards, but their identification is difficult, and the application of appropriate management methods is affected. This study investigates the occurrence and composition of Typlocybinae species in the Greek vineyards. The combination of molecular and morphological analysis identified the species *Arboridia adanae*, *Asymmetrasca decedens*, *Hebata decipiens*, *Hebata vitis*, *Jacobiasca lybica* and *Zygina rhamni*. At the same time, the results of the DNA analysis based on the mitochondrial region revealed many haplotypes within the six different collected species.

**Abstract:**

Insects of the subfamily Typhlocybinae (*Hemiptera*: *Cicadellidae*) are pests of economically important agricultural and horticultural crops. They damage the plants directly or indirectly by transmitting plant pathogens, resulting in significant yield loss. Several leafhoppers of this subfamily use vines as hosts. Accurate and rapid identification is the key to their successful management. The aim of this study is to determine the *Typhlocybinae* species that exist in vineyards all over Greece and investigate the relationship between them. For this purpose, yellow sticky traps were placed, morphological and molecular data were collected, and phylogenetic models were analyzed. The mitochondrial marker Cytochrome Oxidase Subunit I (mt*COI*) was applied for the DNA and phylogenetic analysis. The combination of morphological and molecular data resulted in identifying the existence of six different species all over Greece: *Arboridia adanae*, *Asymmetrasca decedens*, *Hebata decipiens*, *Hebata vitis*, *Jacobiasca lybica* and *Zygina rhamni*. Forty-eight different haplotypes were found to exist in the different regions of the country.

## 1. Introduction

The subfamily *Typhlocybinae* (*Cicadomorpha*: *Cicadellidae*) consists of small, tiny leafhoppers that feed on the contents of leaf parenchyma cells of their host plants [1]. This subfamily includes about 5078 described species worldwide, and it is the second largest cicadellid subfamily after Deltocephalinae [2,3]. Many typhlocybine species are economically important agricultural pests, damaging a variety of *cultivated* crop plant species through direct feeding on the contents of parenchyma cells and spreading plant pathogens [4,5,6,7].

Leafhopper species of the subfamily *Typhlocybinae* are included among the most common insect pests of vineyards that substantially affect grape growth and productivity. Several species are reported to infest vineyards of the Mediterranean basin and worldwide where vines are cultivated. *Arboridia adanae* (Dlabola, 1957), *Asymmetrasca decedens* (Paoli, 1932), *Hebata* (Alboneurasca) *decipiens* Paoli, 1930, *Hebata* (Signatasca) *vitis* (Göthe, 1875), *Erasmoneura vulnerata* (Fitch, 1851), *Jacobiasca lybica* (Bergevin and Zanon, 1922) and *Zygina rhamni* (Ferrari, 1882) are included in the species that very often are reported damaging vineyards [8,9,10,11,12,13,14].

Most *Typhlocybinae* species complete two or three generations per year, and they usually overwinter on plants of the neighboring vineyard vegetation. Nymphs and adults feed on green plant tissues by sucking the leaf parenchyma cell contents reducing the photosynthetic rate [15]. Heavily infested leaves dry out, which has a major consequence on the reduction in the sugar content in the berries [11,16,17,18,19,20,21]. Moreover, some species are vectors of viruses, viroids or phytoplasmas, which are devastating pathogens of vines, and they greatly reduce grape production [22,23,24,25,26,27,28]. Consequently, the estimation of the economic impact of pest infestation and the control practices that must be implemented requires accurate knowledge of the *Typhlocybinae* fauna in vineyards.

Currently, the morphological and molecular data that are collected from phylogenetic studies aim to contribute to a better understanding of the genetics and evolution of different species or within populations of the same species, their biology and ecology, and differences or similarities in their behavior [29,30]. Several developed molecular markers have been used to determine population genetic diversity and genetic variation [31,32,33]. Moreover, the proper identification of those species is mainly based on the morphological characters of male genitalia, and when only nymphs or female adults are found, their identification is problematic. Since many *Typhlocybinae* leafhopper species might coexist in vineyards, scientists are investigating the molecular identity of these species, with the aim of correctly determining the species and consequently providing the farmers with the appropriate pest management system.

The availability of fast DNA sequencing techniques, in combination with robust statistical software, provides accurate solutions in this direction [34]. Mitochondrial genes (mt*DNA*) have been widely used as genetic markers due to their features, such as maternal inheritance, the absence of recombination and a rapid rate of evolution [35]. Mitochondrial DNA is considered to be one of the most accurate molecular detection biomarkers for species differentiation, and barcoding analysis can be used to investigate phylogenetic relationships at the genus level and to illuminate systematic biology [36]. The well-studied mitochondrial gene encoding cytochrome oxidase subunit I (COI) has been preferred in many phylogenetic studies, and it is considered the universal barcode for insect species identification [37,38,39].

Demichelis et al. (2010) described the occurrence of six Empoascini species (*A. decedens*, *Hebata affinis*, *H. decipiens*, *Hebata pteridis*, *H. vitis* and *J. lybica*) based on morphological data isozyme patterns and mt*COI* gene sequences [40]. The genitalic characters of the male seem to be enough to categorize systematically a *Typhlocybinae* individual if it belongs to the referring species. At the same time, *J. lybica* was clearly different from the remaining five species. The genetic distances and bootstrap values clearly discriminate the six species, while *H. vitis* and *A. decedens* are separated together from all the others. In general, the sequences seem to describe the same probable phylogenetic relationships among the Empoascini species studied. 

Lu et al. (2021) strongly support the monophyly of *Typhlocybinae,* considering the most taxonomically comprehensive phylogenetic analyses [41]. Moreover, high bootstrap numbers supported that the currently recognized tribes Alebrini, Empoascini, Dikraneurini and Erythroneurini are also monophyletic. They also describe that the wing characters that are traditionally used to distinguish tribes may not be reliable for this subfamily. Thus, other morphological and molecular characteristics need to be considered in revising the higher classification of this large and diverse group. Moreover, these data support the capability of the male’s genitalia as an important characteristic for Typlocybinae discrimination. When different similar species and life stages are found on a crop, the combination of data collected after morphological and molecular analysis is the safest path for drawing conclusions and making inferences for the proper species identification. 

The aim of this study is to gain information about the species of *Typhlocybinae* leafhoppers that exist in vineyards all over Greece and investigate the possible relationship between them. For this purpose, numeric, morphological and molecular data were collected two years in a row, and different statistical and phylogenetic models were analyzed so as to better understand their presence and origin.

## 2. Materials and Methods

### 2.1. Sampling

In the framework of the National Survey Program regarding quarantine, Cicadomorpha pests and insect vectors of Grapevine flavescence doree phytoplasma in grapes vineyards were surveyed with yellow sticky traps. Two traps were placed in each vineyard surveyed for a period of ten days in different areas of Greece from July to October of 2017 and 2018. In 2017, 160 traps were placed in 80 different vineyards of 34 regional units all over Greece. The corresponding numbers for the year 2018 were 74 traps, 37 vineyards and 21 regional units. After their dispatch, they were transferred to our lab, and the total trapped insects were counted. Those individuals that belong to the subfamily *Typhlocybinae* macroscopically and which were in good condition for further examination were removed from the traps with small forceps, conserved in tubes with ethanol 98% and finally stored at −20 °C (Table S1).

### 2.2. Morphological Identification

For morphological identification of *Typhlocybinae* species, only the male insects were used, and dissection of the males’ genitalia was carried out. Analytically, the abdomen was cut and treated in 10% KOH for 24 h. Then, the internal, unnecessary structures were removed, and male genitalia were mounted on a slide and observed using a microscope. Their characteristics were used for identification to species level, using appropriate and specialized taxonomic keys and illustrations [29,42]. Photographs were taken with an EVOS XL Core imaging system. The rest of the insect body was stored at 98% ethanol for future analysis. Finally, the wing venation of specimens was also observed as an auxiliary character to strengthen the identification result [43].

### 2.3. Faunistic Analysis

The insect species collected were categorized using the criteria of dominance and frequency [44]. The dominance index (Di) represents the percentage contribution of each species on total catches. It was calculated as Di = (ni × 100)/N, where ni = Number of individuals of the species I, and N = Total individuals of all species. A taxon is classified as ‘dominant’, ‘influent’ or ‘recedent’ if it constitutes more than 10%, from 5% to 10%, or less than 5% of the total number of individuals, respectively. The frequency (F) provided information about the distribution of one species in the sampled area. It was calculated as follows: F = (Gi × 100)/S, where Gi = the number of sample records for the species I, and S = the number of all samples. Three categories are recognized about the frequency of occurrence of an insect taxon in the samples. A taxon is classified as ‘constant’, ‘accessory’ or ‘accidental’, if it occurs in more than 50%, from 25% to 50%, or less than 25% of the total number of samples, respectively.

### 2.4. Molecular Analysis

About 30% of the total number of male individuals that were successfully identified at the species level was forwarded for molecular analysis. Every single insect was left on a filter paper until the ethanol was completely removed and the sample was fully dried. Total DNA was extracted using the DNeasy Blood & Tissue Kit (QIAGEN, Hilden, Germany) according to the manufacturer’s protocol, and the final collected volume of the DNA was 20 μL.

One set of primers, LEP-F1 (5′-ATTCAACCAATCATAAAGATATTGG-3′) and LEP-R1 (5′-TAAACTTCTGGATGTCCAAAAAATCA-3′), was used during the Polymerase Chain Reaction. This primer set targets the genes of Cytochrome Oxidase I (COI), which are capable of investigating further the species’ discrimination and the potential phylogenetic variability of the insect population [38,45]. Each PCR reaction mixture contained 5 µL of 10 × PCR buffer, 1.5 µL of MgCl2 (50 mM), 0.5 µL of dNTPs (10 mM), 1 µL of each primer (10 µM), 5 µL of template DNA (20–40 ng), 0.5 μL of the thermostable Taq DNA polymerase (Platinum, Invitrogen, Waltham, MA, USA) and molecular grade water (up to 50 µL). The thermocycling program included an initial denaturation of 3 min at 94 °C, followed by 5 cycles of 94 °C for 30 s, 45 °C for 30 s and 72 °C for 1 min, and by 35 cycles of 94 °C for 30 s, 51 °C for 1 min and 72 °C for 1 min and a final step of extension at 72 °C for 10 min.

The template’s amplification was confirmed by using 5 μL of the PCR products on 1.2% agarose gel electrophoresis, which finally resulted in the observation of an expected 658 bp product. The remaining volume of 45 μL was purified according to the supplier’s instructions of the NucleoFast 96 PCR Clean-up kit (Macherey-Nagel GmbH & Co. KG, Düren, Germany) and then forwarded to Macrogen Europe (Amsterdam, The Netherlands) for automated sequencing analysis. The obtained sequencing results were optimized, generated and aligned through the software Geneious Prime 2023.0.1 (https://www.geneious.com/). The produced mtDNA sequences were checked for their authenticity at the genus or species level according to the BLAST public interface of the National Center for Biotechnology Information (https://blast.ncbi.nlm.nih.gov, accessed on 1 March 2023). The sequences were truncated to 658 bp.

Phylogenetic analysis was carried out using the DnaSP 6.0 software [46] and the software Mega 11.0 [47], which provides a wide choice of models to be applied. Specifically, the factors that were estimated were the guanine–cytosine content (G + C), the average evolutionary divergence (pairwise genetic distance) (d ± SE), the number of variable sites (Vs), the average number of nucleotide differences (k) and the number of haplotypes (h).

Neighbor-Joining, minimum evolution, maximum parsimony, unweighted pair group method (UPGMA) and Jukes–Cantor model analyses were conducted for each species separately, including 1000 bootstrap replicates [48]. The phylogenetic tree was constructed using the neighbor-joining (NJ) method with confidence in the branches (bootstrap) of 1000 replicates in Geneious Prime 2023.0.1. A reference sequence of *Typhlocybinae* (MF938898) was retrieved from the NCBI database and was included as the out-group for the construction of the phylogenetic tree [49]. 

## 3. Results

### 3.1. Sampling

**Figure 1:**
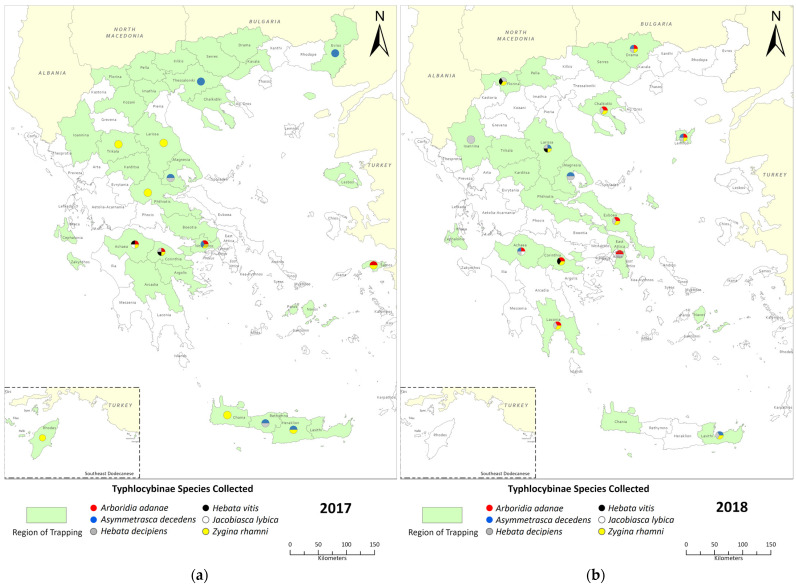
Map of Greece depicting with green color the regions where traps were placed for the years (**a**) 2017 and (**b**) 2018. Each circle refers to which *Typhlocybinae* species were trapped in the corresponding vineyards qualitatively and with different colors. In uncolored regions, no traps were placed, while in areas without circles, no *Typhlocybinae* individuals were collected (designed with the software ArcGIS Pro, V 3.0.3, https://www.esri.com/en-us/arcgis/, accessed on 3 February 2023).

Individuals of *Typhlocybinae* were trapped in 24 and 20 vineyards in 2017 and 2018, correspondingly (Figure 1, Table S1). On the whole, a few Cicadellidae insects were trapped, and it is worth noting that none of them belongs to the vector of Grapevine Flavescence doree phytoplasma.

### 3.2. Morphological Identification

The morphological identification revealed the presence of six *Typhlocybinae* species in the investigated vineyards all over Greece: *Arboridia (Arboridia) adanae* (Dlabola, 1957) (AA), *Asymmetrasca decedens* (Paoli, 1932) (AD), *Hebata* (Alboneurasca) *decipiens* Paoli, 1930 (HD), *Hebata (Signatasca) vitis* (Göthe, 1875) (HV), *Jacobiasca lybica* (Bergevin & Zanon, 1922) (JL) and *Zygina (Zygina) rhamni* Ferrari, 1882 (ZR) (Figure 1).

**Figure 2:**
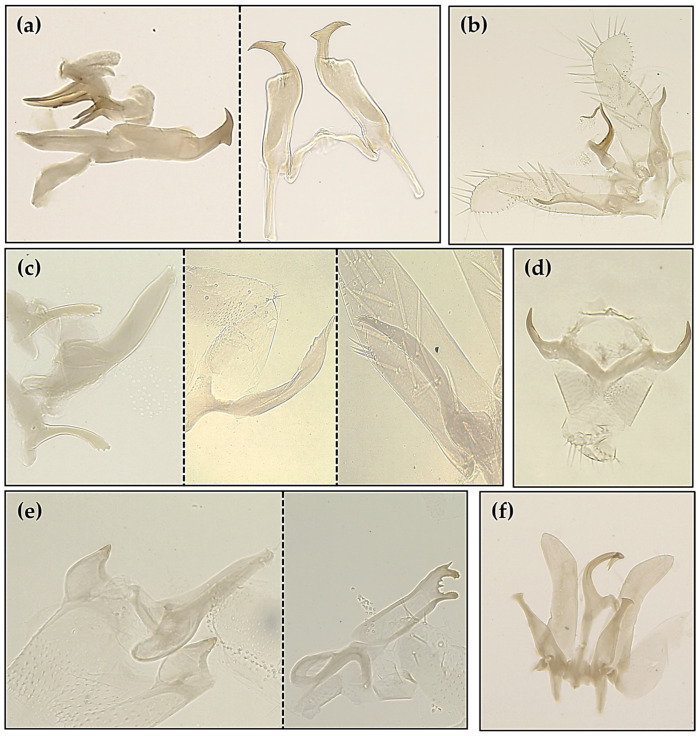
Parts of male genitalia as dissected and photographed under microscope, for the six *Typhlocybinae* species collected: (**a**) *A. adanae* (AA) (aedeagus and styli); (**b**) *A. decedens* (AD) (aedeagus, connective, styli and subgenital plates); (**c**) *H. decipiens* (HD) (aedeagus, anal tube process, pygofer process and stylus); (**d**) *H. vitis* (HV) (anal tube process); (**e**) *J. lybica* (JL) (aedeagus, anal tube process and pygofer appendage); (**f**) *Z. rhamni* (ZR) (aedeagus, connective, styli and subgenital plates).

The identification of the species was based on specific morphological characteristics described in the keys [29,42]. Parts of male genitalia are depicted in Figure 2, with details for all six species (Figure S1).

In some regions, such as Cephalonia, Karditsa, Naxos, Pella and Serres, the insects were completely absent from the traps. On the other hand, the typhlocybine entomofauna consisted of four different species found at Achaea and Corinthia in 2017 and at Drama, Larissa, Lemnos and Achaea in 2018. In the case of *J. lybica*, individuals were collected only in the region of Achaea for both 2017 and 2018. It seems that *Z. rhamni* existed in most vineyards of Greece in 2017, while *H. decipiens* was found in most vineyards in 2018. *Z. rhamni* was present in nine regional units during 2017, while in 2018, *H. decipiens* was collected in thirteen units, which actually covers all sampling regions with the presence of insects, except for Corinthia. Two more *Typhlocybinae* species, *A. decedens* and *A. adanae*, cover a wide range of the Greek area (Figure 1, Table S1).

### 3.3. Faunistic Analysis

The most abundant species was *A. decedens*, which accounted for 31% of the identified individuals (Table S1). According to the criteria of dominance, it was classified as dominant. *A. adanae*, *Z. rhamni* and *H. decipiens* were also classified as dominant, while *H. vitis* and *J. lybica* were classified as influent and recedent, respectively. Concerning the frequency criteria, all the identified species were classified as accidental (Table 1).

### 3.4. Molecular Analysis

Two hundred and eighteen sequences were finally produced from the corresponding number of insects, which were found to belong to six different species based on the morphological results. The analyzed insects were collected in different geographical regions so as to conclude the most equable results. The sequences obtained for the 48 different haplotypes were deposited in GenBank, receiving the accession numbers shown in Table 2. For *A. adanae* and *Z. rhamni*, these are the first sequences for the specific gene area and length that are deposited. All the other species’ sequences were matched to their given names or synonyms. In total, there were 65 variable sites, and the average base composition was A (26.5%), C (15.7%), G (15.7%) and T (42.1%). The maximum value of the pairwise genetic distance was determined between individuals of *A. adanae* and *A. decedens,* and it was equal to 0.379, while the minimum value (0.000) was observed between the samples of the same species. The average value was 0.186.

**Figure 3:**
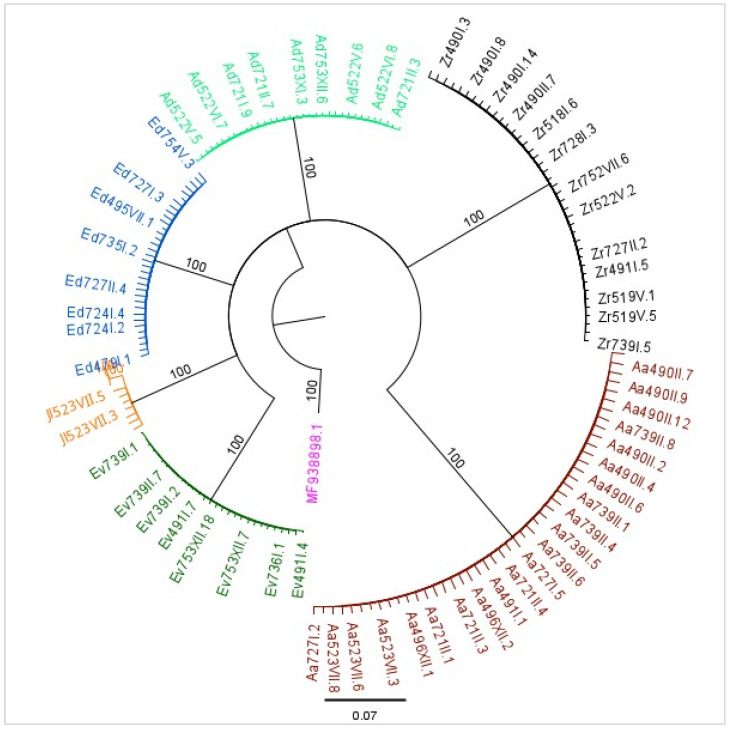
Phylogenetic tree constructed according to the Tamura-Nei genetic model and the Neighbor-Joining building method with bootstrap of 1000 and a reference sequence of *Typhlocybinae* in pink (MF938898). The six different species that were recorded are grouped separately in clades colored differently: *A. adanae* in brown, *Z. rhamni* in black, *A. decedens* in light green, *H. decipiens* in blue, *J. lybica* in yellow and *H. vitis* in green.

The genetic distance of the different haplotypes ranged from 0.001 to 0.227, with an average of 0.010 per species. They also give a clear view of species discrimination, but there is no significant variability between their haplotypes (Table 2). The different models that were applied to the molecular data concluded similar data results, which is clearly shown in the phylogenetic tree constructed (Figure 3).

## 4. Discussion

In the present study, the morphological and molecular data are combined in order to determine which *Typhlocybinae* species are common in the Greek vineyards and investigate their diversity. In total, six species were finally collected and identified as *Arboridia adanae*, *Asymmetrasca decedens*, *Hebata decipiens*, *Hebata vitis*, *Jacobiasca lybica* and *Zygina rhamni*. Among these six species, four were trapped in the majority of the collected sites, although they had different distributions during the two years. These are *H. decipiens*, *Z. rhamni*, *A. decedens* and *A. adanae*. On the other hand, *J. lybica* was observed in only one region, Achaea, for both years, while in the regions of Cephalonia, Karditsa, Naxos, Pella and Serres, no individuals were collected two years in a row.

In the last few years, several species of *Typhlocybinae* have become a serious threat to viticulture, affecting vineyards either directly by their feeding activity on leaves or indirectly by transmission of many plant pathogens. However, few studies about their diversity in vine agroecosystems have been conducted. Rodrigues et al. (2023) studied the presence of Cicadomorpha in 35 vineyards in Portugal and recorded 11 *Typhlocybinae* species, but only 8 of them were identified at the species level [50]. The species recorded were *Alebra coryli*, *H. vitis*, *Fruticidia bisignata*, *J. lybica*, *Ribautiana tenerrima*, *Z. lunaris*, *Z. ordinaria* and *Zyginidia scutellaris*. However, *H. vitis* was by far the most abundant species. Moreover, Duso et al. (2020) carried out a study in four vineyards in Northern Italy and found that the dominant *Typhlocybinae* species were *Erasmoneura vulnerata*, *H. vitis* and *Z. rhamni* [11]. The latter two species were also reported as the most abundant in a vineyard in northeastern Italy [51]. In all the aforementioned studies, *H. vitis* was the most abundant species. *Zygina rhamni* was also abundant only in Italy, while it was absent in Portugal. *Hebata vitis* and *Z. rhamni* were also recorded in our study, but they were not the most populated. Moreover, the most abundant species of the present study, *A. adanae* and *A. decedens,* were absent in Portugal, and they are not reported as abundant in Italy. Several factors affect the insect species’ diversity and abundance [52,53,54]. These include climatic factors, temperature and humidity, latitude and altitude, and plant fauna diversity. It is considered that the general habitat quality plays a great role in the composition of leafhoppers’ population in an area. Thus, the different meteorological conditions and each region’s pattern of biodiversity between the different sampling vineyards are expected to influence the entomofauna that is collected and the complex species that survive in them. The application of insecticides during the trapping period might have hampered insect trapping. Consequently, the fact that no *Typhlocybinae* species were collected in some regions and samplings does not necessarily mean that the insects do not exist in these specific vineyards. A more dedicated sampling model could give a more comprehensive conclusion in the future.

In our study, all specimens belonging to each of the six species were concentrated in separate clades, as is displayed with the six different colors (Figure 3), indicating that the analysis of sequences is a powerful tool for species identification. The molecular analysis and the number of sequences deposited to the nucleotide databases worldwide refer to a genus, a tribe or even a subfamily level; determining the differences between individuals requires a more in-depth analysis. The NCBI database includes a few sequences of the *Typhlocybinae* species, but no insect material was collected in Greece. In the case of *A. adanae* and *Z. rhamni*, no sequences for the specific gene area and length have been deposited before.

Overall, six *Typhlocybinae* species seem to infest the vineyards in Greece, with no specific geographical occurrence. There is no clear evidence that some species prefer to host vineyards between the mainland or the islands or between north Greece instead of the south. Considering that subfamily *Typhlocybinae* is a highly diverse group, some other molecular markers could be investigated in order to further examine the species and population diversity, covering more regions of the country, increasing the number of the samplings, and applying more dedicated research, simultaneously.

## 5. Conclusions

In conclusion, this study includes faunistic, morphological and molecular data for the insects of the subfamily Typhlocibinae collected in vineyards all over Greece. Six different species were found to colonize these vineyards: *Arboridia adanae*, *Asymmetrasca decedens*, *Hebata decipiens*, *Hebata vitis*, *Jacobiasca lybica* and *Zygina rhamni*, with different distribution all over the geographical region of the country (islands, mainland, etc.). The 48 different haplotypes that were deposited in the molecular database give a range of information for other scientists, especially for species with small or no number of sequences included in the NCBI database.

## Figures and Tables

**Table 1:** Dominance and frequency of the *Typhlocybinae* species detected in vineyards of Greece.

	AA	AD	HD	HV	JL	ZR
Dominance	27	31	12	9	2	19
Frequency	6	7	9	4	1	11

**Table 2:** Summary of molecular analysis of mt*COI* gene from different populations for the six *Typhlocybinae* species (*A. adanae* (AA), *A. decedens* (AD), *H. decipiens* (HD), *H. vitis* (HV), *J. lybica* (JL) and *Z. rhamni* (ZR)) and for their different Haplotypes (Haps).

Species	# Seq	G + C	d ± SE	Vs	k	h	GenBank Coding
AA	47	0.344	0.011 ± 0.003	15	6.68270	15	OQ389577-OQ389591
AD	34	0.311	0.003 ± 0.001	4	1.83957	4	OQ381252-OQ381255
HD	34	0.288	0.008 ± 0.003	12	5.06595	10	OQ381258-OQ381267
HV	29	0.316	0.001 ± 0.001	2	0.70936	3	OQ381269-OQ381271
JL	10	0.280	0.014 ± 0.004	16	8.55556	6	OQ381272-OQ381277
ZR	64	0.309	0.003 ± 0.001	5	1.68974	10	OQ389567-OQ389576
Haps	48	0.312	0.171 ± 0.012	237	100.85018	48	-

## Data Availability

Sequencing data of Arboridia adanae, Asymmetrasca decedens, Empoasca decipiens (Hebata decipiens), Empoasca vitis (Hebata vitis), Jacobiasca lybica and Zygina rhamni were submitted to the NCBI Genbank database under the accession numbers OQ389577-OQ389591, OQ381252-OQ381255, OQ381258-OQ381267, OQ381269-OQ381271, OQ381272-OQ381277 and OQ389567-OQ389576, correspondingly.

## Supplementary Materials

The following supporting information can be downloaded at: https://www.mdpi.com/article/10.3390/insects14110894/s1, Table S1: Data presenting the different positions and time of sampling during the years 2017 and 2018, and enumeration data collected during the experimental procedure; Figure S1: Male genitalia as extracted from individuals of *A. decedens* (AD) and *Z. rhamni* (ZR), that were collected in the different regions of Greece.
