# Supplementary material for: Insights into the Diversity and Population Structure of Predominant Typhlocybinae Species Existing in Vineyards in Greece
Source: Insects. 2023 Nov 19;14(11):894. doi: 10.3390/insects14110894 (PMC10672024; doi:10.3390/insects14110894)
Supplement: Supplementary file 1 [file insects-14-00894-s001.zip › insects-2671813-supplementary materials.pdf]

Table S1. Data presenting the different positions and time of sampling during the years 2017 and 2018, and enumeration data collected during the experimental procedure.

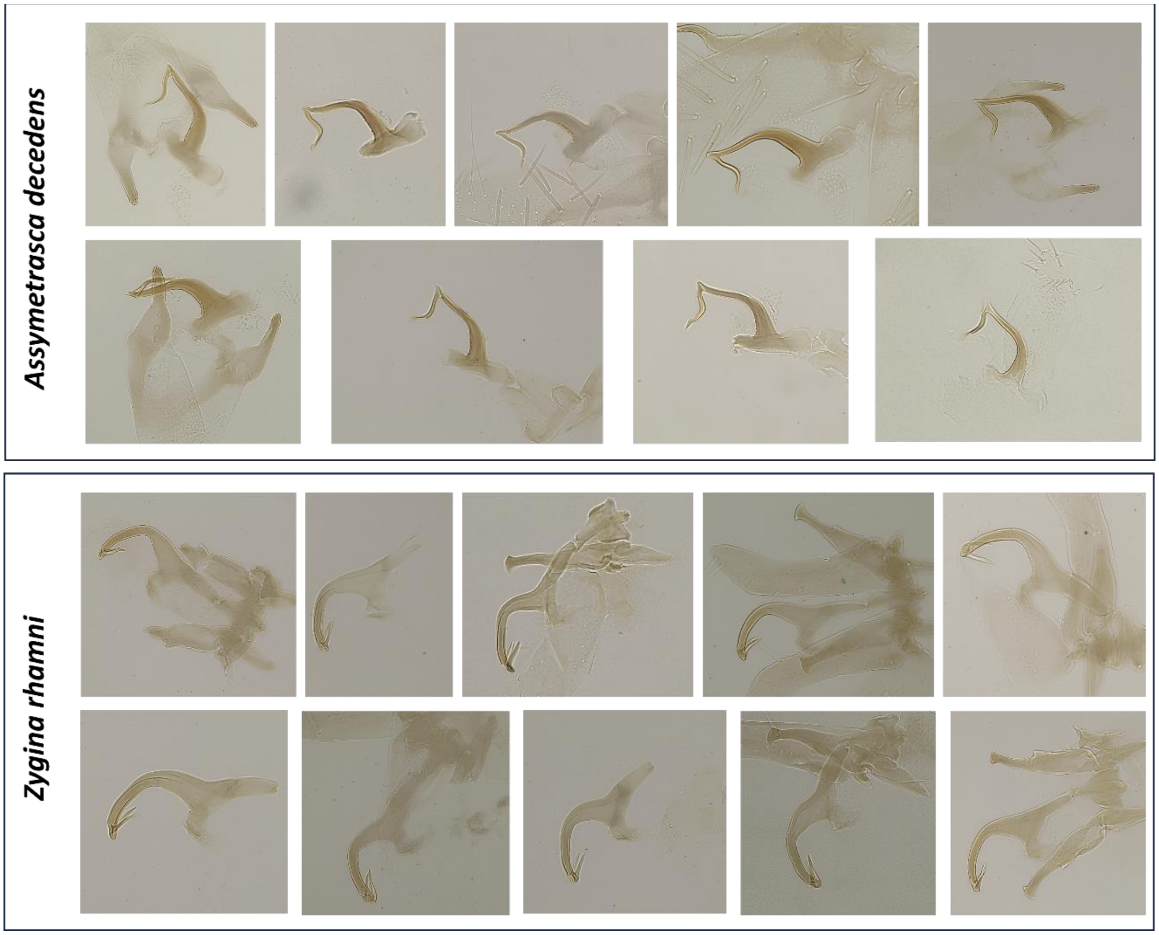

Figure S1. Male genitalia as extracted from individuals of *A. decedens* (**AD**) and *Z. rhamni* (**ZR)**, that were collected in the different regions of Greece.
